# Genetic map of *Triticum turgidum* based on a hexaploid wheat population without genetic recombination for D genome

**DOI:** 10.1186/1471-2156-13-69

**Published:** 2012-08-13

**Authors:** Li Zhang, Jiang-Tao Luo, Ming Hao, Lian-Quan Zhang, Zhong-Wei Yuan, Ze-Hong Yan, Ya-Xi Liu, Bo Zhang, Bao-Long Liu, Chun-Ji Liu, Huai-Gang Zhang, You-Liang Zheng, Deng-Cai Liu

**Affiliations:** 1 Triticeae Research Institute, Sichuan Agricultural University, Wenjiang, Chengdu, Sichuan, 611130, P.R. China; 2 Key Laboratory of Adaptation and Evolution of Plateau Biota, Northwest Institute of Plateau Biology, Chinese Academy of Sciences, Xining, 810001, P.R. China; 3 CSIRO Plant Industry, 306 Carmody Road, St Lucia, QLD, 4067, Australia

**Keywords:** Allopolyploid, Crossability, Doubled haploid, Segregation distortion

## Abstract

**Background:**

A synthetic doubled-haploid hexaploid wheat population, SynDH1, derived from the spontaneous chromosome doubling of triploid F_1_ hybrid plants obtained from the cross of hybrids *Triticum turgidum* ssp. *durum* line Langdon (LDN) and ssp. *turgidum* line AS313, with *Aegilops tauschii* ssp. *tauschii* accession AS60, was previously constructed. SynDH1 is a tetraploidization-hexaploid doubled haploid (DH) population because it contains recombinant A and B chromosomes from two different *T. turgidum* genotypes, while all the D chromosomes from *Ae. tauschii* are homogenous across the whole population. This paper reports the construction of a genetic map using this population.

**Results:**

Of the 606 markers used to assemble the genetic map, 588 (97%) were assigned to linkage groups. These included 513 Diversity Arrays Technology (DArT) markers, 72 simple sequence repeat (SSR), one insertion site-based polymorphism (ISBP), and two high-molecular-weight glutenin subunit (HMW-GS) markers. These markers were assigned to the 14 chromosomes, covering 2048.79 cM, with a mean distance of 3.48 cM between adjacent markers. This map showed good coverage of the A and B genome chromosomes, apart from 3A, 5A, 6A, and 4B. Compared with previously reported maps, most shared markers showed highly consistent orders. This map was successfully used to identify five quantitative trait loci (QTL), including two for spikelet number on chromosomes 7A and 5B, two for spike length on 7A and 3B, and one for 1000-grain weight on 4B. However, differences in crossability QTL between the two *T. turgidum* parents may explain the segregation distortion regions on chromosomes 1A, 3B, and 6B.

**Conclusions:**

A genetic map of *T. turgidum* including 588 markers was constructed using a synthetic doubled haploid (SynDH) hexaploid wheat population. Five QTLs for three agronomic traits were identified from this population. However, more markers are needed to increase the density and resolution of this map in the future study.

## Background

Many important crops such as bread wheat, durum wheat, cotton, oat, coffee, and tobacco are allopolyploids. They originated from the merger of two or more distinct but related genomes, by interspecific hybridization and then genome doubling. Because the majority of accessions of ancestral species were not involved in speciation, many of their unique genes may not present in crops [1,2]. Thus unraveling the genes coding for agronomically-important traits in ancestral species is important for crop improvement. The differences in polyploid levels between a crop and its ancestral species may cause differences in gene expression, especially for a quantitative trait locus (QTL). Therefore, genetic populations with the same ploidy level as a given crop are needed to analyze ancestral genes for crop improvement; however their construction is labor-intensive and time-consuming.

We recently developed a method for synthesizing doubled haploid (SynDH) populations specific for allopolyploid species [3]. *Triticum turgidum* L. (2n = 4x = 28, AABB) and *Aegilops tauschii* Coss. (2n = 2x = 14, DD), the two ancestral species of common wheat (*Triticum aestivum* L., 2n = 6x = 42, AABBDD), were used to demonstrate the method. Unlike existing methods for producing DH populations, this method eliminates the need for *in vitro* culture for extracting haploids and/or chemical treatment for chromosome doubling. Moreover, genetic recombination at the hexaploid level can be restricted to the D genome (thus called diploidization-hexaploid SynDH) or A and B genomes (tetraploidization-hexaploid SynDH), making genetic analysis simpler [3]. However, the effects of applying such a strategy to the construction of genetic maps are not clear.

The objectives of this study were to (i) develop a genetic map of the A and B genomes based on DArT (Diversity Arrays Technology), SSR (simple sequence repeat), ISBP (insertion site–based polymorphism), and high-molecular-weight glutenin subunit (HMW-GS) markers using a tetraploidization-hexaploid SynDH population, (ii) assess the extent of segregation distortion, (iii) evaluate the quality of linkage maps constructed using this strategy by comparing with previously reported maps, and (iv) identify QTLs in this population.

## Results

### Construction of genetic map and QTL analysis

Although this SynDH population was derived from the cross of three parents (LDN/AS313//AS60), all of the D chromosomes came from the single genotype AS60. Thus, segregation of this population was restricted to the A or B chromosomes, which came from the two different tetraploid parents, *T. turgidum* AS313 and LDN. Therefore, polymorphic markers between the two *T. turgidum* parents can be used in genetic mapping. A total of 606 polymorphic markers between *T. turgidum* AS313 and LDN were identified, including 521 DArT, 81 SSR, two ISBP, and two HMW-GS markers. These markers were used to genotype the SynDH population.

**Figure 1:**
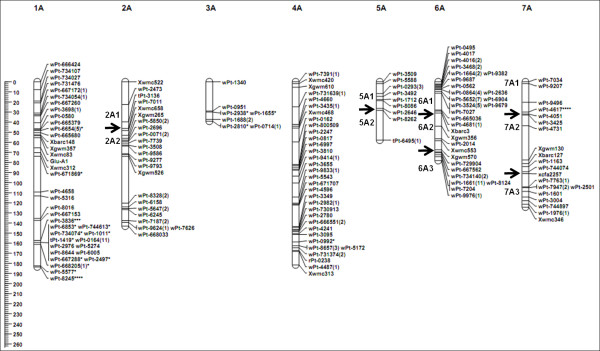
**Genetic linkage map of A genomes obtained using 113 doubled-haploid lines from the AS313/LDN//AS60 population.** Numbers in parentheses indicate the number of markers that are not present at each locus. Segregation distortion of markers are indicated by asterisks at significance levels of 0.05 (*), 0.01 (**), 0.005 (***), 0.001 (****), 0.0005 (*****), and 0.0001 (******). The black arrow indicates the point of separation between two different linkage groups. The scales on the left indicate distances in centiMorgans (Kosambi).

**Figure 2:**
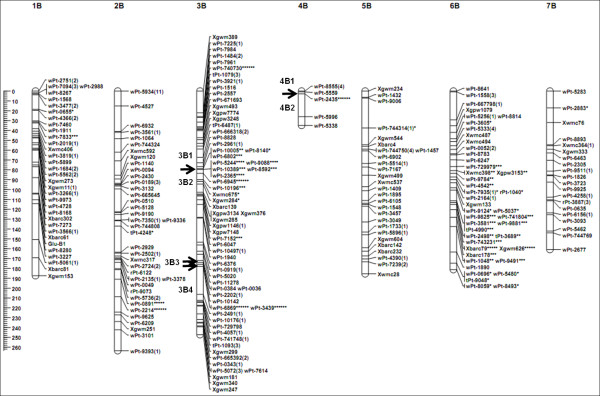
**Genetic linkage map of B genomes obtained using 113 doubled haploid lines from the AS313/LDN//AS60 population.** Numbers in parentheses indicate the number of markers that are not present at each locus. Segregation distortion of markers are indicated by asterisks at significance levels of 0.05 (*), 0.01 (**), 0.005 (***), 0.001 (****), 0.0005 (*****), and 0.0001 (******). The black arrow indicates the point of separation between two different linkage groups. The scales on the left indicate distances in centiMorgans (Kosambi).

Of the 606 markers, 588 (97%) were included in the genetic map. They included 513 DArT (98%), 72 SSR (89%), one ISBP, and two HMW-GS markers (Table 1). These markers were assigned to 24 linkage groups on 14 chromosomes, giving a total map length of 2048.79 cM (Table 1). This map showed good coverage of the A and B genome chromosomes, apart from 3A, 5A, 6A, and 4B (Figure 1, 2). Map length of the A genome (796.18 cM) was shorter than that of the B genome (1252.61 cM). Chromosomes 1A, 3A, 4A, 1B, 2B, 5B, 6B, and 7B were each represented by a single linkage group, while 2A, 5A, 6A, 7A, 3B, and 4B were each represented by two or more linkage groups (Figure 1, 2). The average length of these linkage groups was 146.34 cM, with a maximum of 263.48 cM, for 2B, and a minimum of 32.90 cM, for 4B. The average distance between markers on these linkage groups ranged from 1.12 cM for 6A to 5.95 cM for 7B. The overall average was 3.48 cM between any two markers across the linkage map (Table 1).

**Table 1:** Marker distribution on A- and B-genome chromosomes

**Chromosome**	**Length (cM)**	**Number of target markers**							**Average length cM/marker**	Mean density**
		**SSR**	**ISBP**	**DArT**			**Glutelin**	**Total**		
				**wPt**	**tPt**	**rPt**				
1A	183.61	5	-	48	4	0	*Glu-A1*	58 (13)*	3.17	7.06
2A	141.23	4	-	28	2	0	-	34 (0)	4.15	7.06
3A	39.21	0	-	10	0	0	-	10 (3)	3.92	9.80
4A	181.61	4	-	40	0	3	-	47 (1)	3.86	5.86
5A	56.01	0	-	11	2	0	-	13 (0)	4.31	7.00
6A	74.17	4	-	57	4	1	-	66 (0)	1.12	3.53
7A	120.34	4	-	20	0	0	-	24 (1)	5.01	6.69
A genome	796.18	21	-	214	12	4	1	252 (18)	3.16	5.94
1B	187.43	8	-	42	1	0	*Glu-B1*	52 (2)	3.60	6.25
2B	263.48	4	-	54	2	2	-	62 (3)	4.25	7.53
3B	241.31	16	1	71	3	1	-	92 (16)	2.62	4.64
4B	32.9	0	-	9	0	0	-	9 (1)	3.66	8.23
5B	185.53	10	-	26	1	1	-	38 (1)	4.88	7.42
6B	181.18	9	-	43	3	1	-	56 (28)	3.24	6.47
7B	160.78	4	-	22	0	1	-	27 (1)	5.95	8.46
B genome	1252.61	52	1	267	10	6	1	336 (52)	3.73	6.29
Total	2048.79	72	1	481	22	10	2	588 (70)	3.48	6.13

*Number of markers with segregation distortion is shown in parentheses.

**the mean density equal to *L*/*(n-1)* where *n* is the number of unique markers per chromosome length *L*.

A total of five QTLs were identified in this population. Two QTLs, located on chromosomes 7A and 5B, were for spikelet number (Table 2). These two QTLs explained 19.2% and 10.2% of the phenotypic variance, respectively. Two QTLs, on 7A and 3B, for spike length explained 19.3% and 15.1% of the phenotypic variance, respectively. The final QTL, on 4B, for 1000-grain weight explained 14.6% of the variance.

**Table 2:** Putative QTLs detected in the SynDH population

**Trait**	**QTL**	**Marker interval**	**LOD score**	**R**^**2**^**(%)**^**a**^	**Estimated additive effect**^**b**^
Spikelet number	*QSpn.scau-7A*	*wPt-4731- Xgwm130*	6.08	19.22	−0.50
	*QSpn.scau-5B*	*wPt-730009- Xwmc28*	3.32	10.18	0.36
Spike length	*QSl.scau-7A*	*wPt-4731- Xgwm130*	5.53	19.26	−0.49
	*QSl.scau-3B*	*Xgwm389- wPt-7225*	4.48	15.13	−0.44
1000-grain weight	*QTgw.scau-4B*	*wPt-7233- wPt-5559*	3.80	14.58	1.72

^a^ Percentage of phenotypic variation explained by each QTL.

^b^ indicates a positive or negative effect from the AS313 allele.

### Segregation distortion

Of the genotypic data collected in this study, 49.8% of the alleles in the SynDH population were derived from AS313, 46.1% were from LDN, and the remaining 4.1% was missing data. The overall allele ratio between those from AS313 and LDN was not significantly different at a significance level of 5%. However, significant deviation was found for 70 (11.90%) out of the 588 markers. Of these, 28 (4.7%) were in favor of LDN and 42 (7.1%) were in favor of AS313 (Table 1; Figure 1), consisting of seven (9.72%) SSR and 63 (12.9%) DArT markers. These distorted markers were distributed on 11 of the 14 chromosomes (Figure 1, 2; Table 1). Among them, 13 were on chromosome 1A, 16 were on 3B, and 28 were on 6B. All of the distorted markers on 3B favored LDN alleles, while all of the 28 distorted markers on 6B favored AS313 alleles. Of the 13 distorted markers on 1A, ten favored AS313 alleles and the other three (*wPt-6654*, *wPt-5577*, and *wPt-8245*) favored LDN alleles (Figure 1).

### Map comparisons

**Figure 3:**
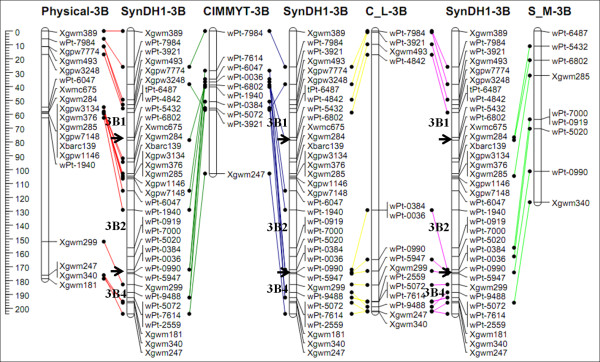
**Comparison of chromosome 3B maps.** The SynDH1 map (SynDH1-3B) obtained in this study was compared with the physical map (physical-3B) [7], the CIMMYT-integrated map (CIMMYT-3B) [4], the durum wheat integrated map (C-L-3B) [5], and triticale genetic map (S-M-3B) [6]. The scales on the left side indicate distances in cM (Kosambi). The black arrow indicates the point of separation between two different linkages. To reduce complexity, only markers shared between these maps are shown. Map comparison was performed using the JoinMap 4.0 program [44]].

To evaluate the quality of the genetic map developed in this study (SynDH1), locations of the shared markers were compared with those in the CIMMYT integrated map (CIMMYT) [4], durum wheat integrated map (C-L) [5], and triticale genetic map (S-M) [6]. Discrepant marker orders were observed in some regions, mostly on chromosomes 1A and 2A. The order of the majority of shared markers was highly consistent among these four linkage maps (see Additional file 1). The comparison of chromosome 3B with the four linkage maps is shown in Figure 3. Furthermore, the genetic map of chromosome 3B from this study was also highly consistent with its physical map (Figure 3) [4-7].

## Discussion

The SynDH1 population used in this study was derived from the interspecific hybridization of *T. turgidum* with *Ae. tauschii*, followed by spontaneous chromosome doubling [3]. We demonstrated in this study that such a population can be effectively used to generate genetic maps. This map has been successfully used to locate high-molecular-weight glutenin subunits on loci *Glu-A1* and *Glu-B1*, and to identify five QTLs of three agronomic traits. However, this study identified as many as 24 linkage groups, which is significantly larger than the number of the 14 haploid chromosomes of the A and B genomes. To reduce the number of the linkage groups, additional markers are needed for a better covered, high-density map.

Low map coverage, duplicated marker loci, or segregation distortion can cause inconsistent marker order in genetic maps [8]. Chromosome rearrangements (such as small translocations, deletions, and inversions) can also result in marker inconsistency [8]. Compared to previously reported CIMMYT integrated map (CIMMYT) [4], durum wheat integrated map (C-L) [5], and triticale genetic map (S-M) [6], discrepant marker orders were observed in some regions, mostly on chromosomes 1A and 2A. According to the marker order in the genetic maps of these two chromosomes (see Additional file 1), these discrepancies appear to have been caused by chromosome inversions. In a recently reported genetic map for triticale, deletion of a fragment of chromosome 1A and translocation of 2A caused discrepancies in marker positions and order across populations [6].

Segregation distortion is a common phenomenon that can be influenced by factors affecting fertility of either gametes or zygotes [9]. Environmental effects also affect segregation distortion and are assumed to influence gametophyte selection [10]. Compared with F_2_ and DH, RILs (recombinant inbred lines) are more prone to segregation distortion because of repeated selective forces [11]. Segregation distortion markers have been reported in common wheat [8,12-15] and its two ancestral species of *T. turgidum*[16-20] and *Ae. tauschii*[21].

If a biological segregation distortion locus exists, the concerned locus and those flanking region would all deviate from the expected Mendelian segregation ratio [22]. Therefore, biological segregation distortion affects a cluster of loci that form a segregation distorted region (SDR). Using the criterion of Paillard *et al*. [13], a SDR should contain at least three closely adjacent loci. Based on this criterion, SDRs were only found on chromosomes 1A, 3B, and 6B in this study (Figure 1, 2). Other distortion loci scattered along the chromosomes 3A, 4A, 7A, 1B, 2B, 4B, 5B, and 7B were likely a result of non-biological factors. The limited number of the SynDH lines used could be one of these factors.

Because all the F_1_ haploid hybrid plants from LDN/AS313//AS60 produced F_2_ doubled haploid plants [3], segregation distortion in the present study was most likely generated in the production process of the haploid hybrids by wide hybridization between *T. turgidum* and *Ae. tauschii.* Because no *in vitro* culture was applied during this interspecific hybridization procedure [3], its involvement in the segregation distortion can be ruled out. The production of conventional haploids via *in vitro* culture may lead to segregation distortion, because the ability of *in vitro* regeneration is genotype-dependent. When crossing *T. turgidum* with *Ae. tauschii*, different alleles of crossability QTL between LDN and AS313 may affect segregation differently. It is known that crossability genes control the ability of crossing between different species, and affect the seed-setting of interspecific crosses [23,24]. Crossability can be promoted by recessive alleles and inhibited by dominant alleles. The inhibiting effect of chromosome 6B [25] and the promoting effect of 3B [26] on crossability in LDN may cause marker segregation favoring AS313 on 6B and LDN on 3B (Figure 2). Because *Xgwm626* and *Xbarc79* on chromosome 6B gave the most severe segregation distortions, and their flanking markers were all less skewed, the crossability QTL may be situated between these two markers. Likewise, a QTL affecting segregation may also exist near *wPt-6945* on 3B. The distorted segregation favoring AS313 near wPt-3836 on 1A suggests that this genotype may have a recessive crossability QTL in this region [27]. These results suggest that the distorted segregation in the SynDH population depends on variations in crossability alleles between the two *T. turgidum* parents. In this regard, genotypic effects on crossability need to be considered when using SynDH populations for genetic mapping.

## Conclusions

A genetic map of *T. turgidum* including 588 markers was constructed using a synthetic doubled haploid (SynDH) hexaploid wheat population, derived from the interspecific hybridization of *T. turgidum* with *Ae. tauschii*, followed by spontaneous chromosome doubling. This map has been successfully used to locate high-molecular-weight glutenin subunits on loci *Glu-A1* and *Glu-B1*, and to identify five QTLs of three agronomic traits. However, more markers are needed to increase the density and resolution of this map in the future study. Moreover, the distorted segregation in the SynDH population may be caused by variations in crossability alleles between the two *T. turgidum* parents. In this regard, genotypic effects on crossability need to be considered when using SynDH populations for genetic mapping.

## Methods

### Plant material

Plant materials used in this study included 113 synthesized doubled haploid (SynDH) lines and their parents, *T. turgidum* ssp. *turgidum* line AS313 and ssp. *durum* line Langdon, and *Ae. tauschii* ssp. *tauschii* accession AS60. The procedure of SynDH was described previously by Zhang *et al.*[3]. Briefly, the F_1_ hybrids between AS313 and LDN were pollinated by AS60. No embryo rescue technique or hormone treatment was applied during this interspecific hybridization procedure. The haploid interspecific hybrids were self-pollinated and SynDHs were produced by spontaneous chromosome doubling with the help of unreduced gametes [3]. This SynDH population has recombinant A and B chromosomes between *T. turgidum* lines AS313 and LDN in a background of non-recombinant D chromosomes from *Ae. tauschii* AS60.

### Field trials and trait evaluation

Field trials were conducted in the crop season of 2010–2011 at the Triticeae Research Institute experimental station in Wenjiang, Sichuan Province, China. Individual plants of the 113 SynDH lines were spaced 10 cm apart within 2 m long rows, and the row spacing was 30 cm. This experiment consisted of two replicates. Five agronomic traits (plant height, tiller number per plant, spike length, spikelet number, and 1000-grain weight) were evaluated from 10 random selected plants from each plot at maturity. Plant height (HT) was calculated as the average height in centimeters measured from the soil surface to the tip of the spike (awns excluded). Spike length (Sl), spikelet number (Spn), and 1000-grain weight were measured on 10 selected spikes from one line (usually the main spike per plant was chosen). Kernels from a sample of 10 spikes from each plot were threshed, counted, and weighed to calculate 1000-grain weight.

### HMW-GS markers

Two high-molecular-weight glutenin subunit (HMW-GS) loci, *Glu-A1* on 1AL and *Glu-B1* on 1BL, were used for mapping [3].

### SSR, ISBP, and DArT genotyping

A bulk leaf sample from five plants for each of the SynDH lines and parents was used for DNA isolation using the 2 × CTAB method [28]. Fifty-nine ISBP (insertion site-based polymorphism) markers [7], and 539 SSR markers including 195 *wmc*[29,30], 159 *gwm*[31], 113 *barc*[32], 49 *gpw*[33], 12 *gdm*[34], 8 *cfd*[35], 2 *cfa*[33], and 1 *psp*[36] were selected to screen the parents. PCR amplifications were performed as described by Zhang *et al*. [3]. The amplified fragments were separated by electrophoresis on 6% polyacrylamide denaturing gels and visualized using the silver-staining method [37].

Genomic DNA from SynDH lines and their parents was sent to Triticarte (Yarralumla, Australia) for profiling using Diversity Arrays Technology (DArT) [38,39]. The common wheat *PstI* (*TaqI*) v3.0 DArT array, which comprises 19,000 clones, was used. Of these clones, about 7000 are known to be polymorphic in a wide range of wheat cultivars, and about 3500 markers have been assigned to chromosomes using nulli-tetrasomic lines derived from Chinese Spring. Hybridization of genomic DNA to the DArT array, image analysis, and polymorphism scoring were carried out as previously described by Akbari *et al*. [40]. DArTs are biallelic dominant markers. Each marker is scored for each sample: 0, 1, or -, where “-” indicates that the marker could not be reliably scored for that sample. DArT calls were converted into “A” (AS313), “B” (LDN), and “-” (missing data) by comparison against parental scores.

### Map construction

Segregation data were analyzed with QTL IciMapping v3.1 [41], and markers were grouped using a logarithm of odds (LOD) >5. The markers within each group were then ordered with RECORD [42] and rippled with SARF (sum of adjacent recombination frequencies). The marker order was used to sort markers within linkage groups and graphical genotypes were examined in Excel 2003 (see Additional file 2). At this step, singletons (a single locus in one progeny line that appears to have recombined with both of its directly-neighboring loci) were replaced by missing values in the dataset and calculations were repeated until no singletons were found. Ungrouped (not corrected data) markers were anchored to previous linkages using a LOD >4.5. All calculations were repeated for new linkage groups. Independent linkages on the same chromosomes with Kosambi distances [43] between subsequent markers below 50 cM were integrated as one linkage group. The χ^2^ analysis, map drawing, and map comparison were performed using the JoinMap 4.0 program [44]. A χ^2^ goodness of fit analysis for each marker was performed to test for deviation from the 1:1 expected segregation ratio in doubled haploids at a 5% level of significance.

### QTL analysis

QTL IciMapping v3.1, based on the inclusive composite interval mapping (ICIM) model [41], was used for QTL analysis. Threshold values were calculated using 1,000 permutations and QTLs were considered real when ICIM showed the presence of a significant peak at a level of 0.05. Estimates of the positions of QTL corresponded to the peak of the ICIM scans. The percentage of phenotypic variation explained by each QTL was calculated with a single factor regression (R^2^).

## Competing interests

The authors declare that they have no competing interests.

## Authors’ contributions

LZ, JTL, and MH performed most of the research and drafted the manuscript. LQZ, ZWY, ZHY, BZ, BLL, and DCL assisted in the collection of genotypic and phenotypic data. YXL assisted in DArT analysis. CJL revised the manuscript. HGZ, YLZ, and DCL supervised and coordinated the study. All authors read and approved the final manuscript.

## Supplementary Material

Additional file 1 **Map comparison of A and B genome chromosomes between SynDH1 (this study) and the CIMMYT-integrated map (CIMMYT)**[4]**, the durum wheat integrated map (C-L)**[5]**, and the triticale genetic map (S-M)**[6]. The scales on the left indicate distances in cM (Kosambi). To reduce complexity, only markers shared between these maps are shown. Map comparison was performed using the JoinMap 4.0 program [44]. Click here for file

Additional file 2 Marker information used for linkage group construction. Click here for file
