# Supplementary figures and images for: Genetic map of Triticum turgidum based on a hexaploid wheat population without genetic recombination for D genome
Source: BMC Genet. 2012 Aug 13;13:69. doi: 10.1186/1471-2156-13-69 (PMC3470960; doi:10.1186/1471-2156-13-69)

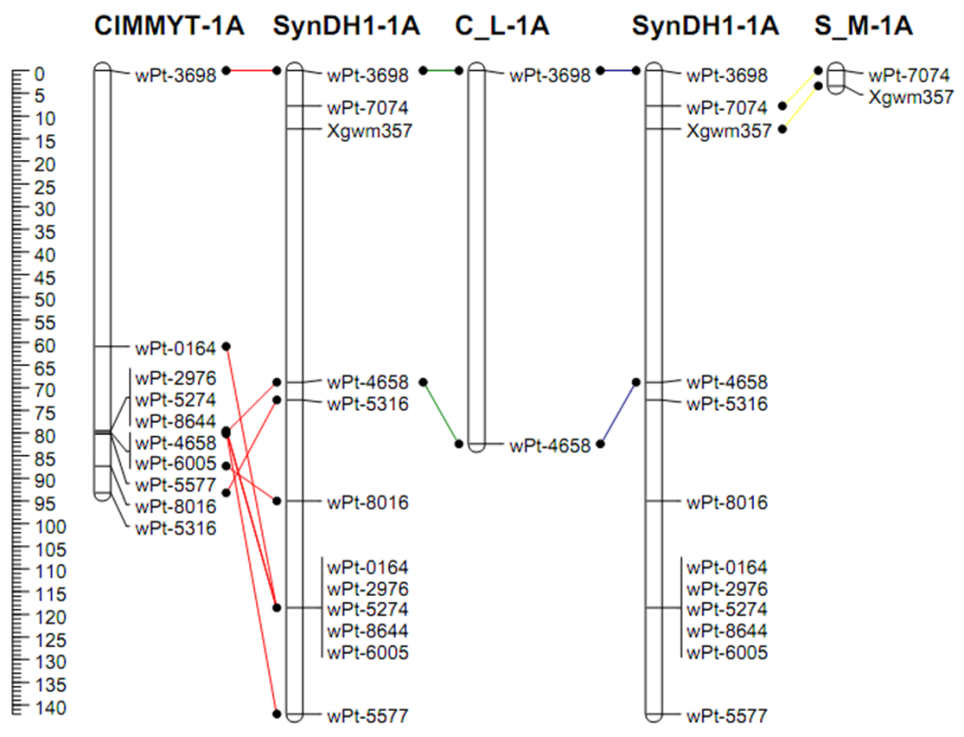

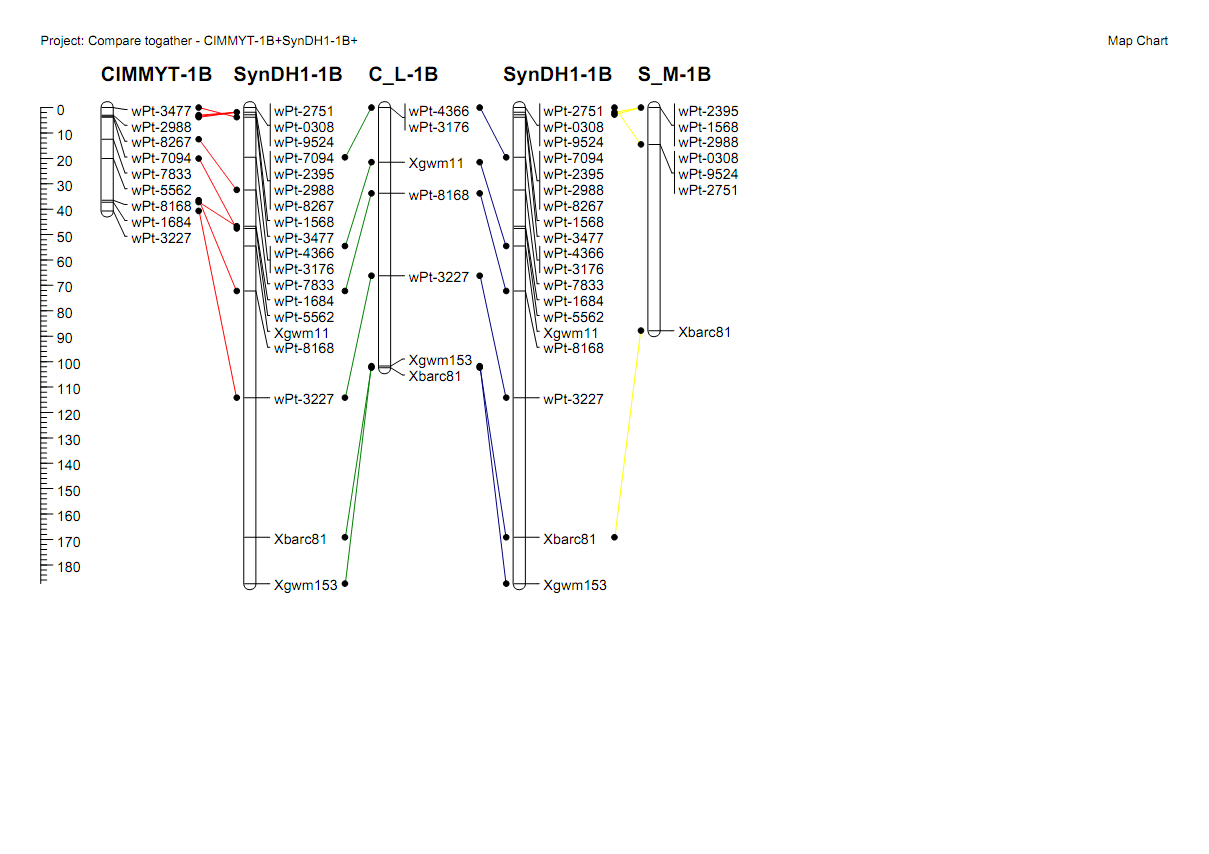


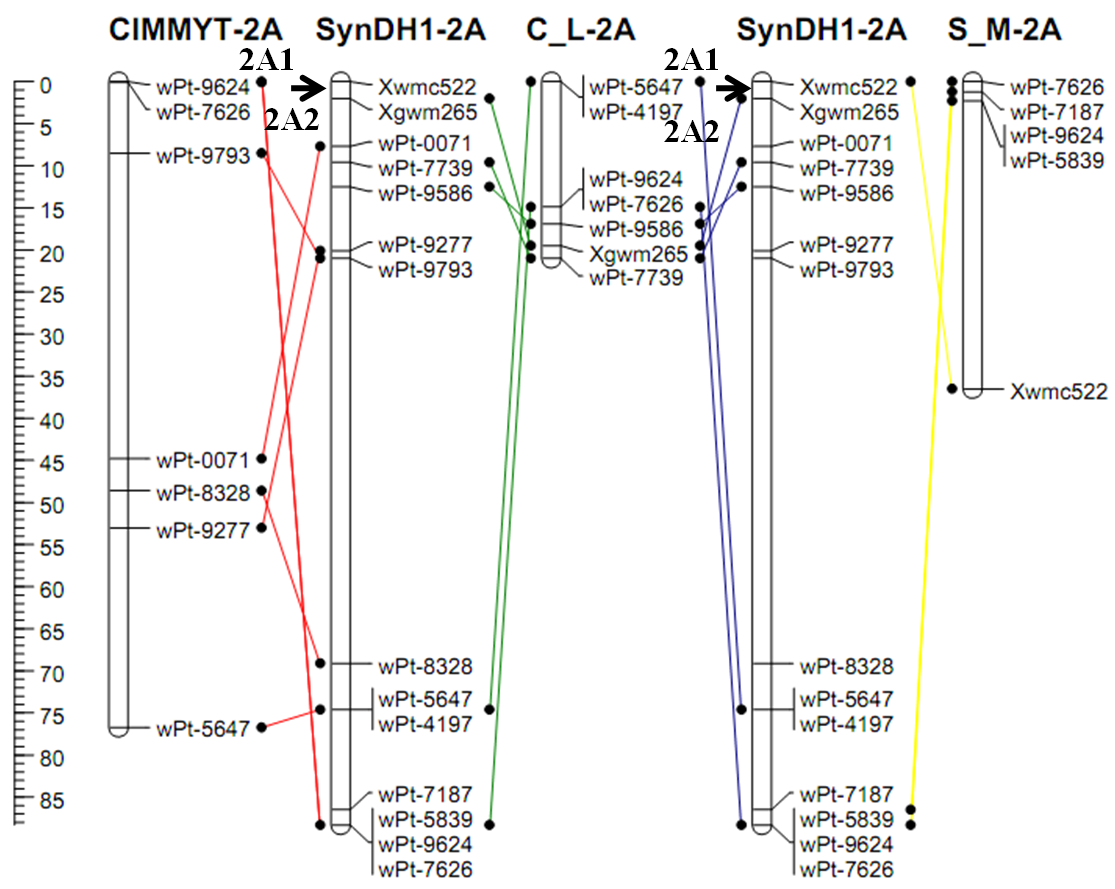

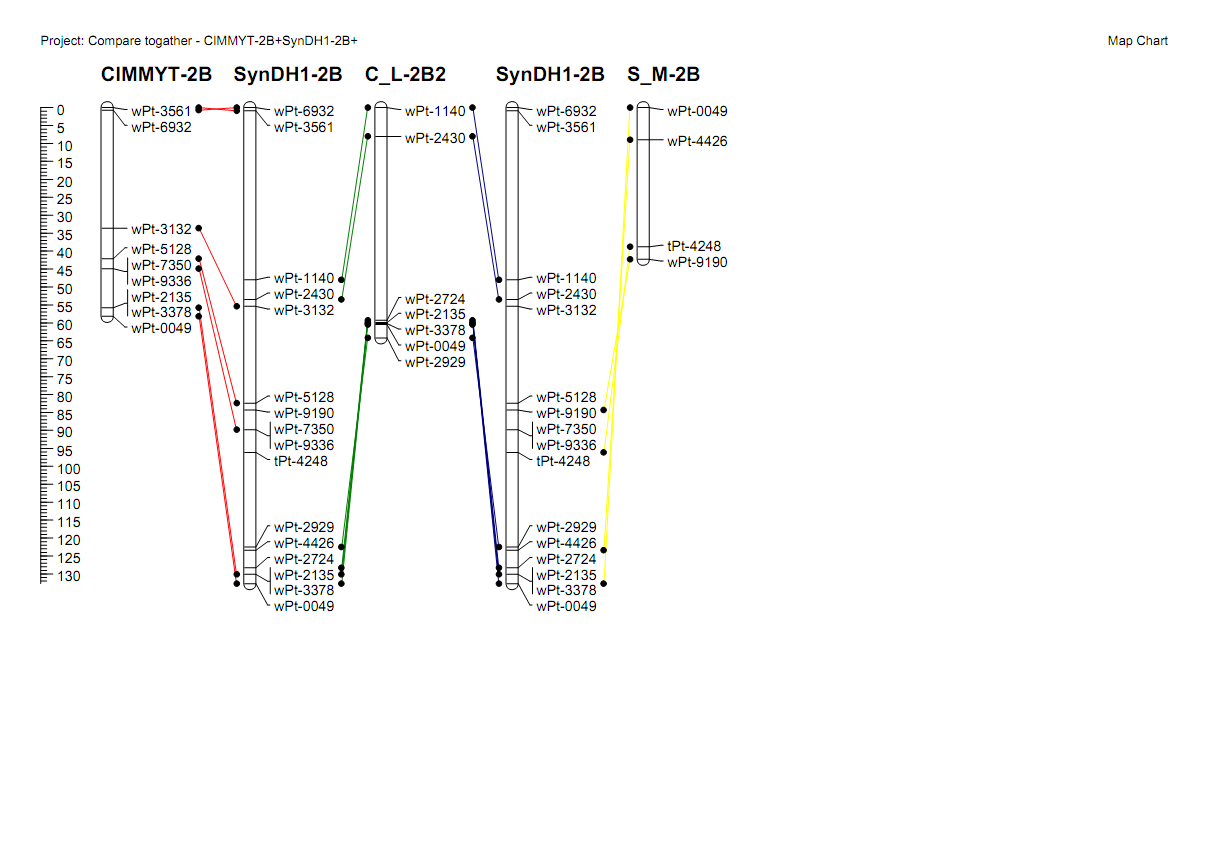


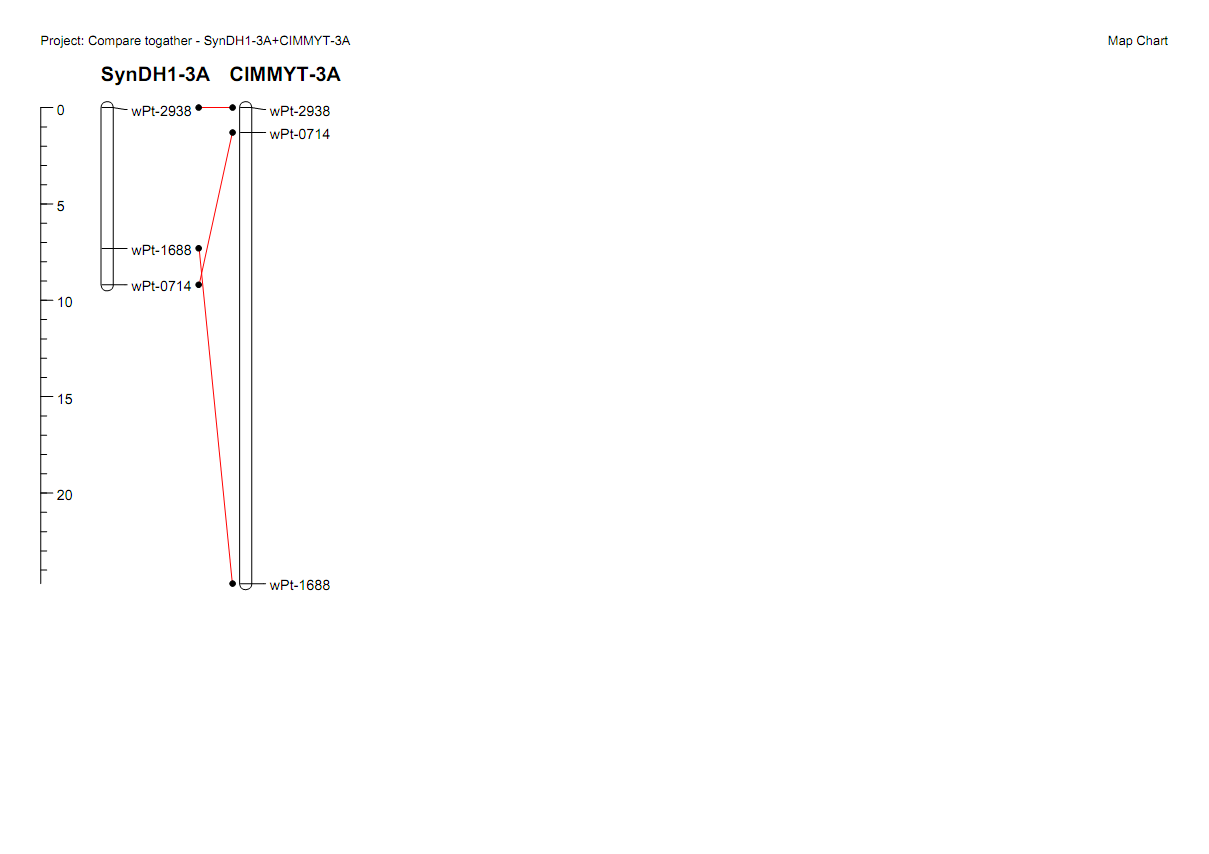


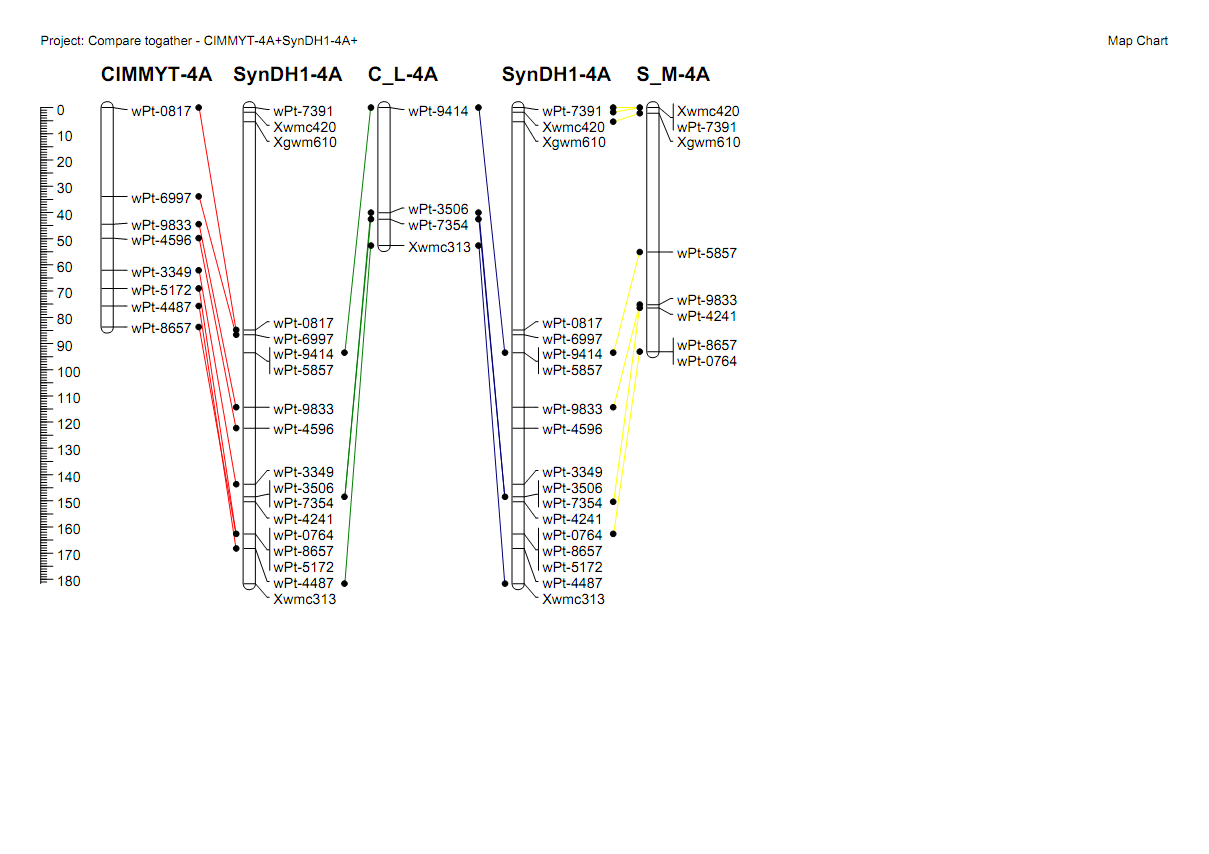

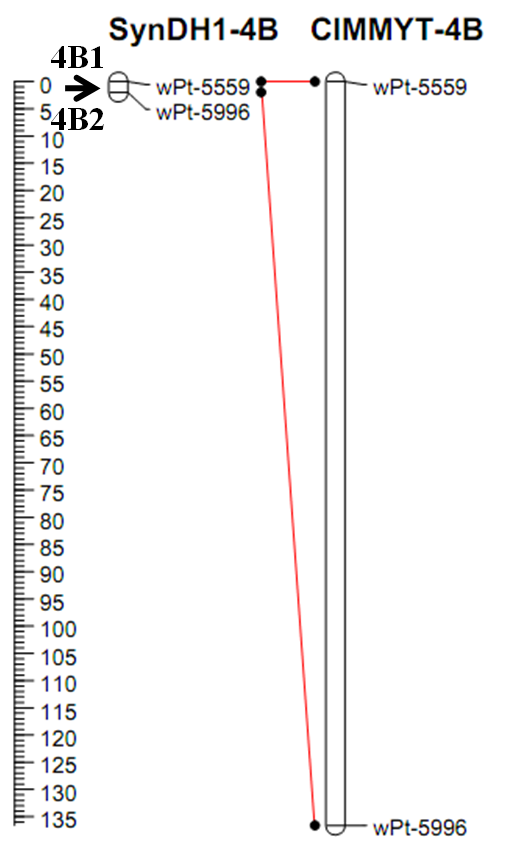


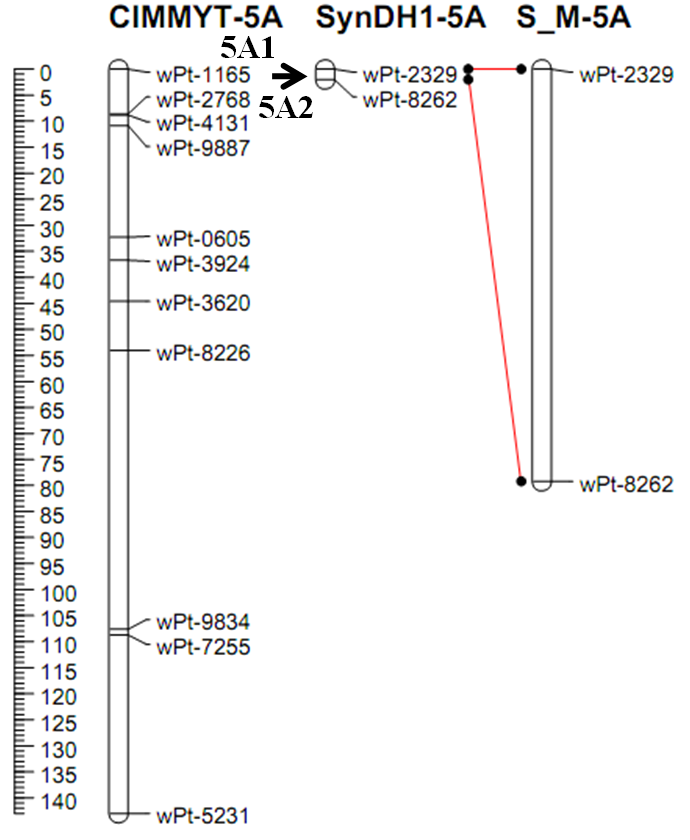

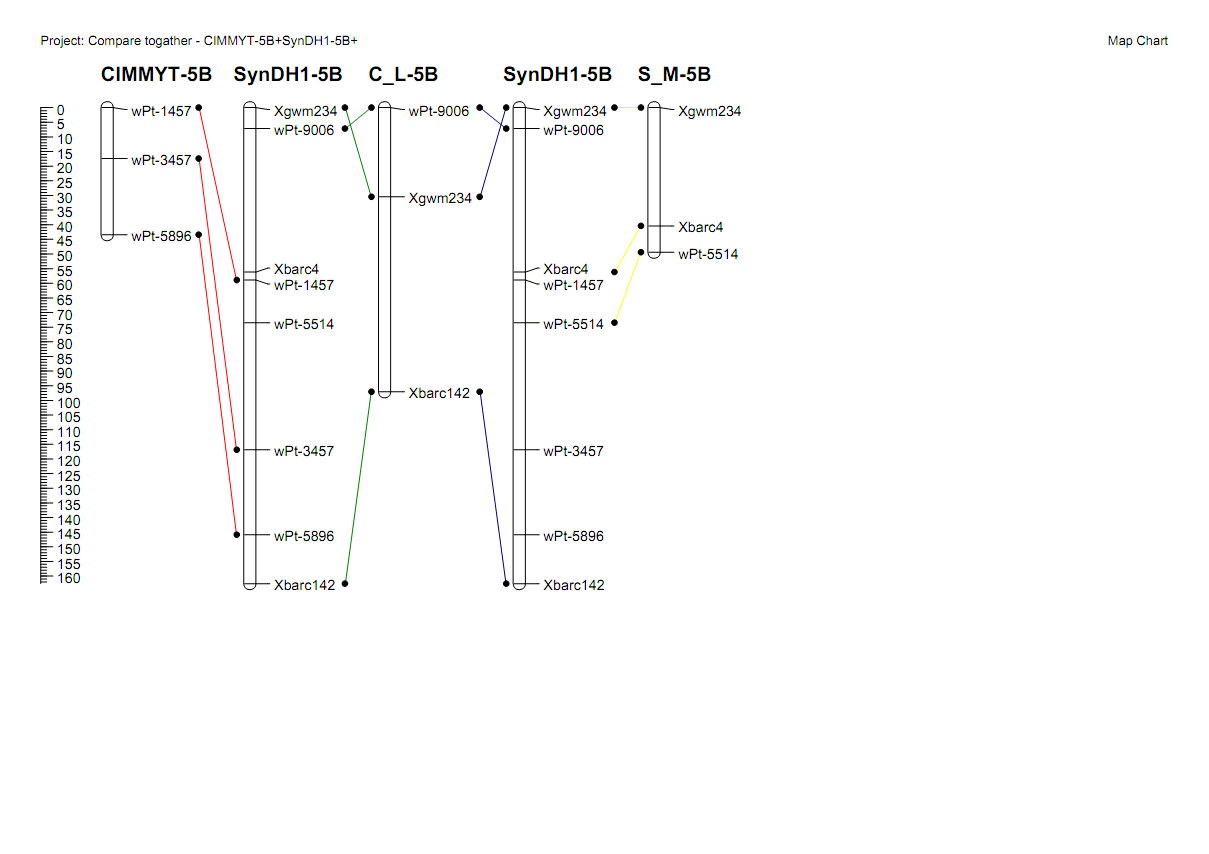


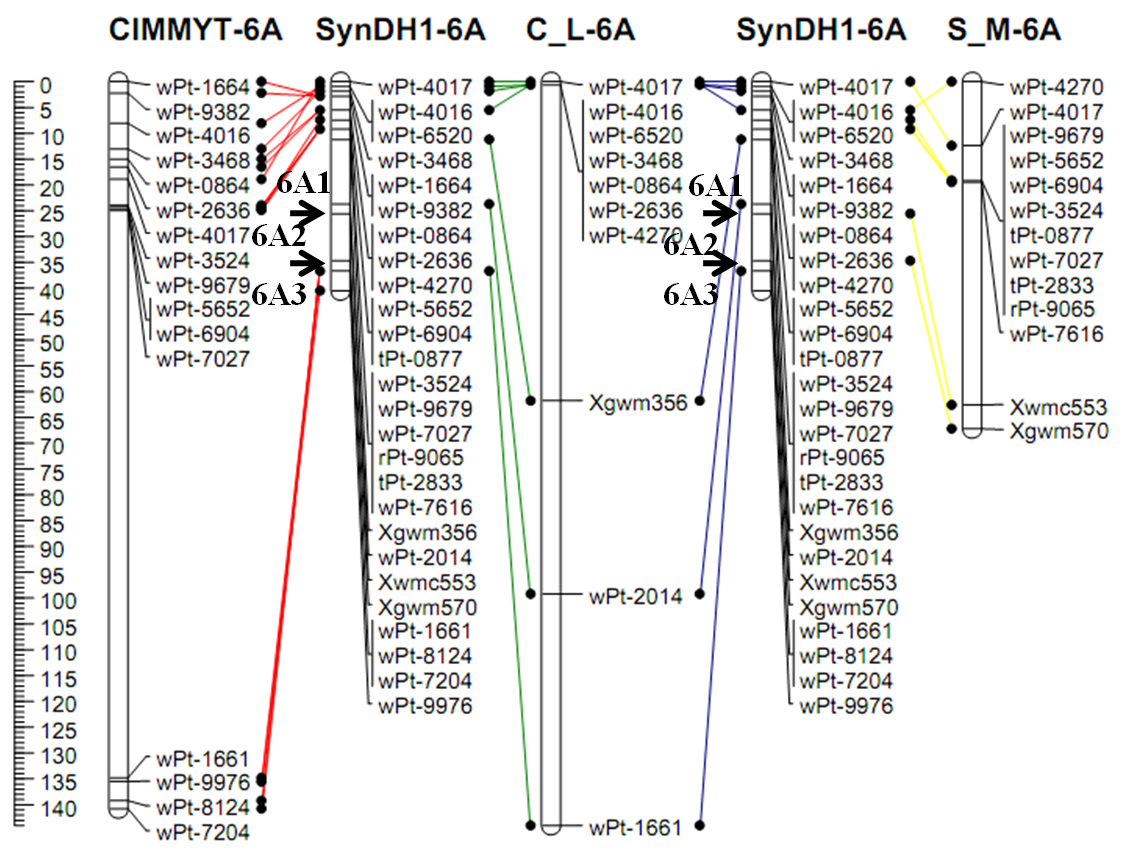

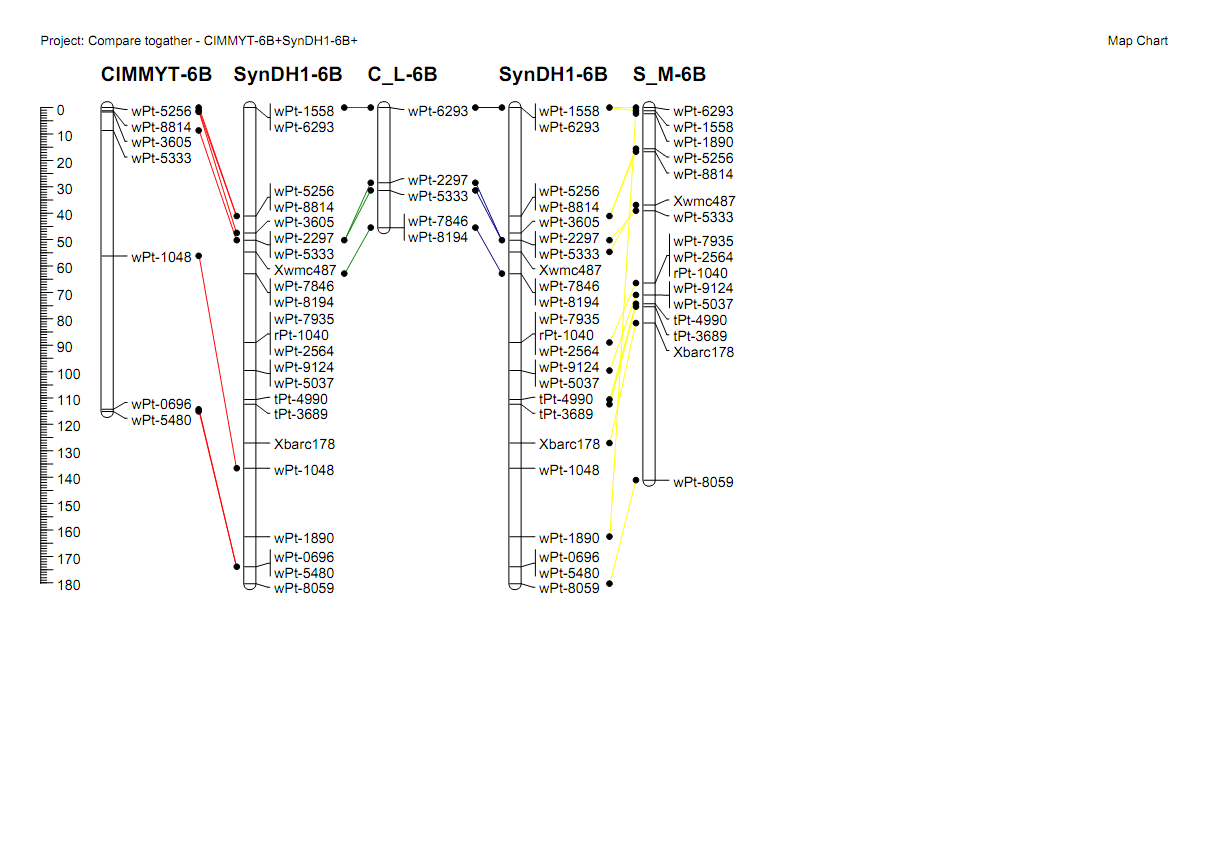


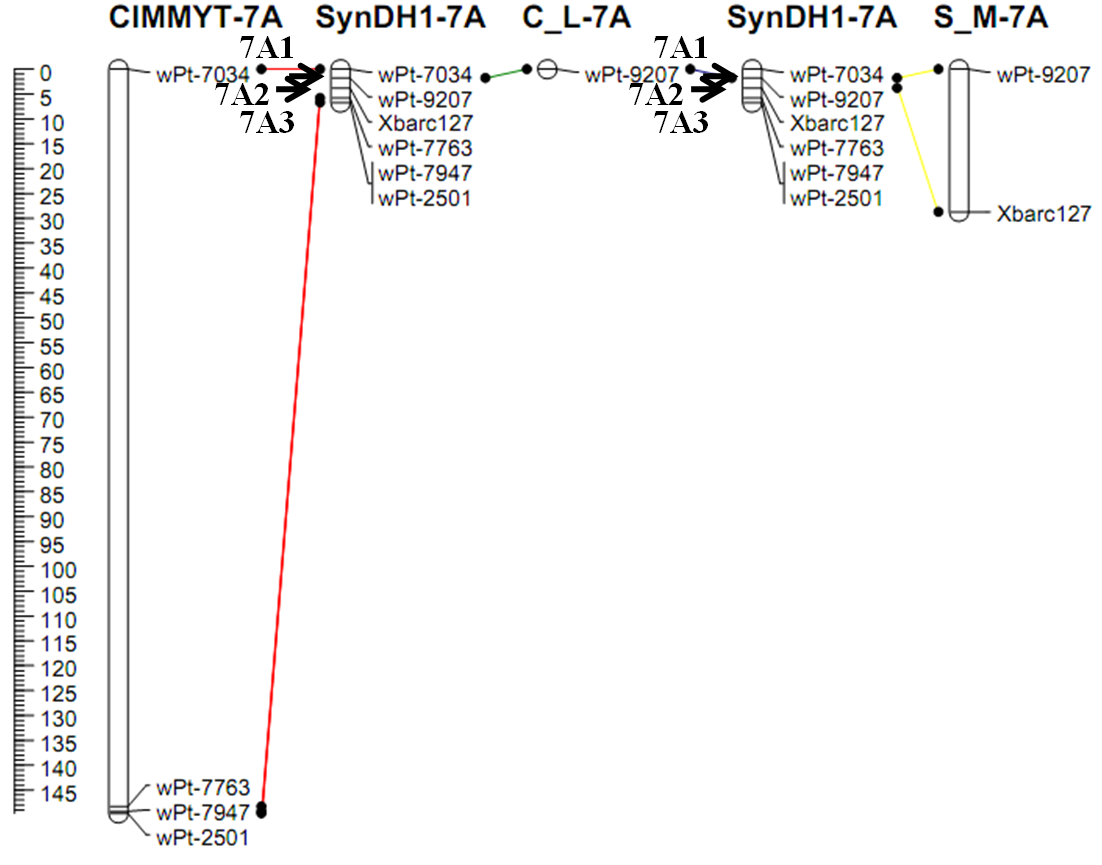

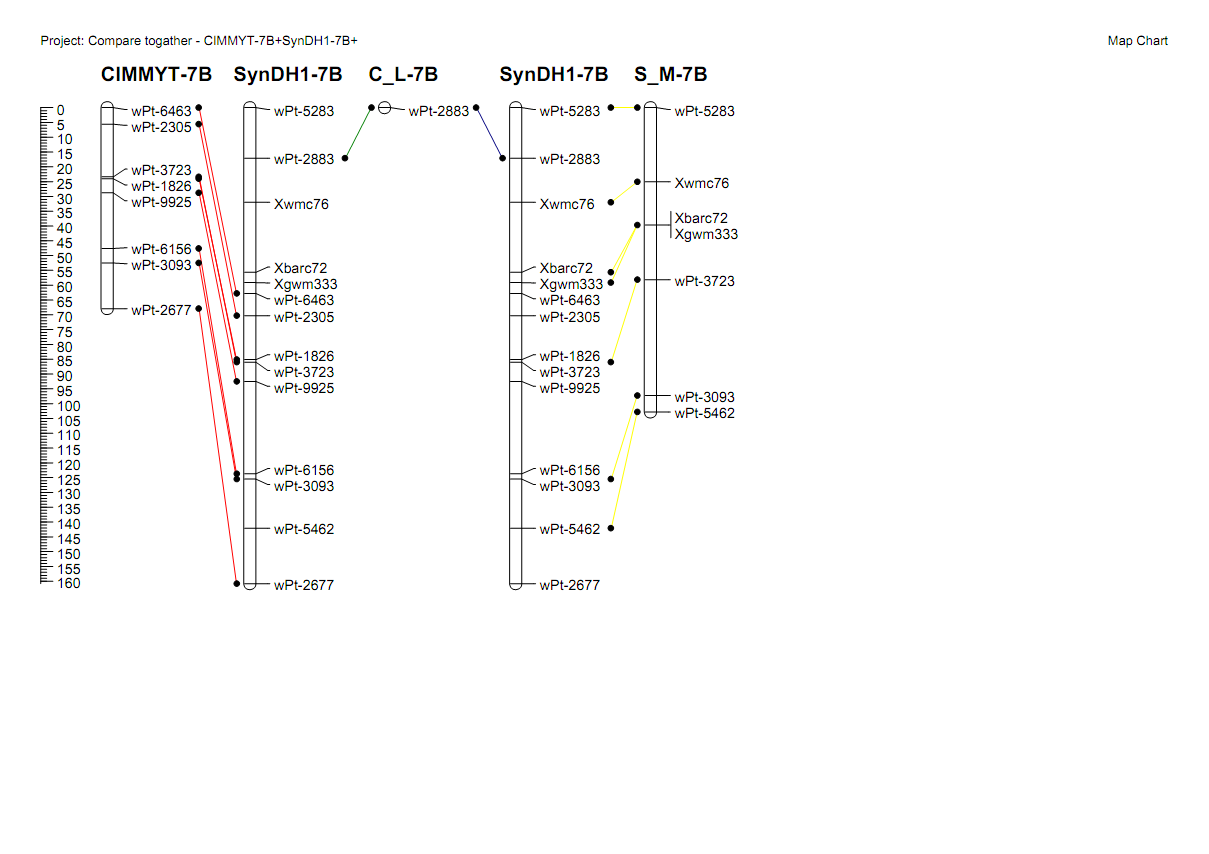

Supplement: Additional file 1 — Map comparison of A and B genome chromosomes between SynDH1 (this study) and the CIMMYT-integrated map (CIMMYT)[4], the durum wheat integrated map (C-L)[5], and the triticale genetic map (S-M)[6]. The scales on the left indicate distances in cM (Kosambi). To reduce complexity, only markers shared between these maps are shown. Map comparison was performed using the JoinMap 4.0 program [44]. [file 1471-2156-13-69-S1.doc]
